# Innovative Multilayer Biodegradable Films of Chitosan and PCL Fibers for Food Packaging

**DOI:** 10.3390/foods14142470

**Published:** 2025-07-14

**Authors:** Justyna Jakubska, Andrzej Hudecki, Dominika Kluska, Paweł Grzybek, Klaudiusz Gołombek, Wojciech Pakieła, Hanna Spałek, Patryk Włodarczyk, Aleksandra Kolano-Burian, Gabriela Dudek

**Affiliations:** 1 Department of Physical Chemistry and Technology of Polymers, Faculty of Chemistry, Silesian University of Technology, Strzody 9, 44-100 Gliwice, Poland; justyna.jakubska@polsl.pl (J.J.); dk301141@student.polsl.pl (D.K.); pawel.grzybek@polsl.pl (P.G.); 2 Department of Physical Chemistry and Technology of Polymers, PhD School, Silesian University of Technology, Akademicka 2A, 44-100 Gliwice, Poland; 3 Lukasiewicz Research Network-Institute of Non-Ferrous Metals, 44-100 Gliwice, Poland; andrzej.hudecki@imn.lukasiewicz.gov.pl (A.H.); hanna.spalek@imn.lukasiewicz.gov.pl (H.S.); patryk.wlodarczyk@imn.lukasiewicz.gov.pl (P.W.); aleksandra.kolano-burian@imn.lukasiewicz.gov.pl (A.K.-B.); 4 Materials Research Laboratory, Faculty of Mechanical Engineering, Silesian University of Technology, Konarskiego 18A, 44-100 Gliwice, Poland; klaudiusz.golombek@polsl.pl (K.G.); wojciech.pakiela@polsl.pl (W.P.)

**Keywords:** biodegradable films, multilayer materials, chitosan, PCL electrospun fibers, food packaging

## Abstract

The growing accumulation of plastic packaging waste poses severe environmental and health challenges. To address these issues, significant research has been devoted to developing biodegradable films; however, their weak mechanical and barrier properties limit their practical utility. This study introduces an innovative multilayer film production method, combining electrospun polycaprolactone (PCL) fibers with a chitosan matrix. Two configurations were investigated: (1) nonwoven PCL layers placed between chitosan sheets and (2) a chitosan sheet sandwiched between two nonwoven PCL layers. Both systems were evaluated using PCL fibers derived from medical-grade and technical-grade polymers. The chitosan/polycaprolactone/chitosan (CH/PCL/CH) configuration demonstrated superior performance, achieving enhanced interlayer cohesion and significantly improved mechanical strength, durability, and barrier properties. Notably, this configuration achieved tensile strength and elongation at break values of 57.1 MPa and 36.3%, respectively—more than double those of pure chitosan films. This breakthrough underscores the potential of multilayered biopolymer films as eco-friendly packaging solutions, offering exceptional promise for sustainable applications in the food packaging industry.

## 1. Introduction

The increasing amount of plastic packaging poses a significant challenge to the modern world. Accumulating in both landfills and aquatic environments, polymer-based films have a negative impact on ecosystems and environmental health. To prevent this problem, many researchers focus on films based on biopolymers. Recently, materials based on polysaccharides have attracted increasing interest. They are characterized by low toxicity, biodegradability, and biocompatibility. However, their use in the packaging industry is limited due to their poor mechanical and hydrophobic properties compared to traditional packaging materials [1]. An important strategy to improve the properties of packaging based on biodegradable polymers is to develop materials consisting of two or more layers. This type of packaging enables the extension of the shelf life of stored food products by enhancing their physical and chemical properties [2,3]. Andrade et al. [4] studied the properties of PLA-PVA multilayer films containing phenolic compounds. These films were obtained by thermocompression, where an inner layer of poly(vinyl alcohol) was laminated with two outer layers of poly(lactic acid). The results showed that all multilayers possessed tensile properties similar to PLA films, with increased stretchability (8–43%). Moreover, the multilayer film barrier’s ability to regulate water vapor and oxygen was higher than that of the monolayer films. Zhang et al. [5] investigated a multilayer film with chitosan as the outer layer and pullulan with clove EO emulsion as the inner barrier layer. The multilayer film showed more favorable mechanical, optical, and hydrophobic properties than the single-layer film. Shai et al. [6] investigated the properties of multilayer films comprising a cellophane coating of PCL and chitosan. The results showed that, compared to the single-layer cellophane film, the tensile strength and Young’s modulus of the multilayer films decreased, indicating that they are softer than the cellophane film. Additionally, they exhibit higher oxygen and water vapor barrier properties.

Recently, fibers formed from electrospinning have increasingly been used as component layers. The main advantages of using electrospun fibers are their good ductility, lightness, and high corrosion resistance, which contribute to the mechanical properties of such materials. Several studies have demonstrated that integrating electrospun layers can significantly improve film performance. For example, Ebrahimzadeh et al. [7] developed a bilayer film composed of electrospun chitosan and poly(vinyl alcohol) fibers on a chitosan substrate, resulting in higher tensile strength and reduced elongation at break compared to monolayer chitosan films. Similarly, Nilsuwan et al. [8] reported enhanced mechanical properties in bilayer films made from electrospun PLA and cast fish gelatin. Malafatti et al. [9] investigated trilayer structures based entirely on electrospun PLA and starch, showing improved plastic deformation and selective cracking, which indicated layered energy dissipation during stretching. Quiles-Carrillo et al. [10] demonstrated that introducing an electrospun PLA layer between extruded PLA layers resulted in improvements in mechanical strength, as well as optical and functional properties. These studies confirm the usefulness of electrospun fibers in multilayer systems; they often rely on synthetic components such as PLA or PVA. Moreover, most of them focus on mechanical performance without exploring alternative fabrication methods that could enhance sustainability or simplify layer integration. Therefore, there is a clear need for innovative approaches that combine biodegradable and bio-based polymers, such as chitosan, more practically and suitably for large-scale use.

In response to these challenges, we propose a novel method for producing composite biodegradable films based on a chitosan matrix combined with polycaprolactone (PCL) fibers obtained via electrospinning. The proposed method differs from those described in the literature, as the final product is formed by thermally welding all layers at the melting point of PCL. Furthermore, the multilayer films are fabricated in two configurations: one involves placing nonwoven PCL layers between chitosan sheets, while the other sandwiches a chitosan sheet between two nonwoven PCL layers. Both configurations were evaluated using PCL fibers obtained from medical-grade and technical-grade polymers to compare the influence of PCL grade, quality, and purity [11]. What is more, since chitosan is typically processed in an acidic environment by using acetic acid, the addition of hydrophobic PCL fibers can help reinforce the material and improve its stability [12]. We hypothesized that this novel method of bonding layers and integrating PCL fibers with chitosan sheets would significantly enhance the films’ properties, particularly their mechanical performance. To evaluate this hypothesis, we analyzed the films’ mechanical, hydrophilic, barrier, structural, optical (transparency), and thermal properties. These results were compared to monolayer chitosan films produced without electrospun PCL fibers.

## 2. Methods and Materials

### 2.1. Materials

Two types of polymeric materials were used to obtain biodegradable PCL fibers: (i) PCL with Mw = 70,000–90,000 from Sigma Aldrich, St. Louis, MO, USA, which was named PCLm, and (ii) PCL of varying chain lengths, from Perstrop, Warrington, UK, for technical applications, which was named PCLt. A combination of solvents was used to dissolve the PCLm and PCLt polymer materials: Chemland’s 99.9% chloroform and Chemland’s 99.9% acetone. For preparing chitosan films, chitosan 30–100 cps (MW = 250,000 g/mol, DD ≥ 90%) was purchased from Sigma-Aldrich (Steinheim, Germany), acetic acid (99.5–99.9%) was delivered from Avantor (Gliwice, Poland), and glycerol was supplied by Merck (Darmstadt, Germany).

### 2.2. PCL Fiber Fabrication

Two separate 70:30 mixtures of chloroform and acetone were prepared for the fabrication of biodegradable PCL fibers. High-purity PCL was added to the first mixture to create a 17% PCLm solution, while low-purity PCL was added to the second mixture to produce a 17% PCLt solution. Both solutions were subjected to an electrostatic field to generate fibers. The fiber formation process was carried out using a flow rate of 10 mL/h through a single nozzle, with an applied voltage ranging from 0.8 to 1.3 kV. The positive electrode consisted of a standard nozzle, while the negative electrode was a rotating drum with a diameter of 219 mm and a length of 700 mm. The process was conducted under controlled conditions at a relative humidity of 34% ± 5% and a temperature of 24 °C ± 3 °C. Electrospinning was performed using a TONG LI TECH TL-BM 700 device, resulting in two types of nonwoven materials: PCLm and PCLt.

### 2.3. Film Preparation

The chitosan film was prepared using a widely recognized solution casting method, as in our previous works [13,14]. This approach enabled the formation of a thin polymer layer, which was ultimately integrated with electrospun nanofibers. Chitosan (2% *w/v*), the main structural component, was dissolved in a 1% *v/v* mixture of acetic acid and water, while 30% *w/w* glycerol was used as a plasticizer.

### 2.4. Combining PCL Nonwovens with Chitosan

The obtained PCLm and PCLt nonwovens were used to fabricate multilayer structures. For this purpose, layers of nonwoven polycaprolactone (PCLm and PCLt) and chitosan (CH) were assembled into the following configurations: (i) PCLt/CH/PCLt, which consists of two outer layers of PCLt enclosing a single CH layer; (ii) PCLm/CH/PCLm, composed of two outer PCLm layers with a central CH layer; (iii) CH/PCLt/CH, featuring two CH layers surrounding a single PCLt layer; and (iv) CH/PCLm/CH, comprising two CH layers enclosing a single PCLm layer.

To fabricate these multilayer structures, 10 × 10 cm sections of nonwoven PCLm, PCLt, and chitosan were cut to size. Additionally, two aluminum foils (20 × 20 cm) were prepared to assist in the layering process. The layers were arranged between the aluminum foils according to the desired configuration.

**Figure 1:**
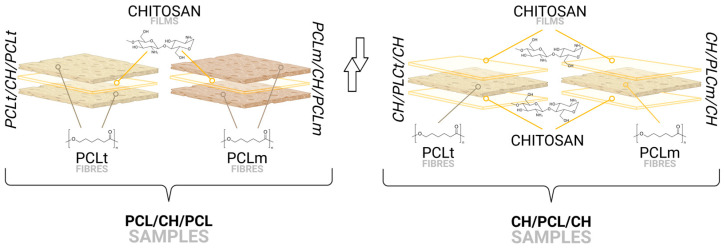
Schematic representation of obtained films’ layer configurations.

The assembled sandwich structures were then thermally treated at 180 °C for 1 min per side, resulting in a total heating time of 2 min per sample. This temperature was selected to prevent the thermal degradation of chitosan while allowing partial melting of PCL, ensuring proper adhesion between layers. After heating, the samples were allowed to cool to room temperature, after which the aluminum foil was gently removed. The final multilayer structures—PCLt/CH/PCLt, PCLm/CH/PCLm, CH/PCLt/CH, and CH/PCLm/CH—were then forwarded for further testing to evaluate their properties and performance (Figure 1).

### 2.5. Mechanical Properties

**Figure 2:**
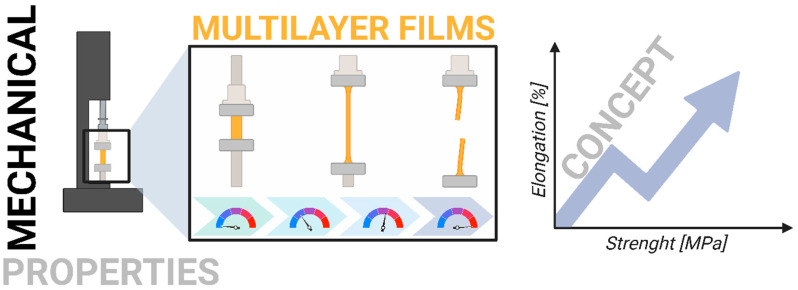
Schematic presentation of research of mechanical properties.

To assess the film’s mechanical properties, tensile strength and elongation at break were analyzed by the ASTM D882 standard [15]. The measurements were conducted using the Instron 4466 apparatus (Figure 2).

### 2.6. Hydrophilic Properties

The hydrophilic properties of the obtained films were determined using a three-step gravimetric method by measuring the moisture content (MC), degree of swelling (SD), and total soluble matter (TSM) of the prepared samples, using the following formulas:

(1) $$MC\left(\%\right)=\frac{\left(M_{1}-M_{2}\right)}{M_{1}}\cdot 100$$

(2) $$SD(\%)=\frac{\left(M_{3}-M_{2}\right)}{M_{2}}\cdot 100$$

(3) $$TSM(\%)=\frac{\left(M_{2}-M_{4}\right)}{M_{2}}\cdot 100$$

where

*M*_1_—mass of sample [g].
*M*_2_—mass of sample after drying at 100 °C for 24 h [g].
*M*_3_—mass of sample after 24 h immersion in 100 mL of distilled water [g].
*M*_4_—mass of sample after re-drying at 100 °C for 24 h [g].

Furthermore, the dynamic water contact angle of the obtained films was measured using a semi-automatic goniometer (OCA15EC, DataPhysic, Filderstadt, Germany). A 5 μL droplet of deionized water was applied to the film surface, and measurements were taken immediately, after 30 s, and after 60 s. Next, the contact angle was calculated using the average of ten different measurements.

### 2.7. Gas Permeability

**Figure 3:**
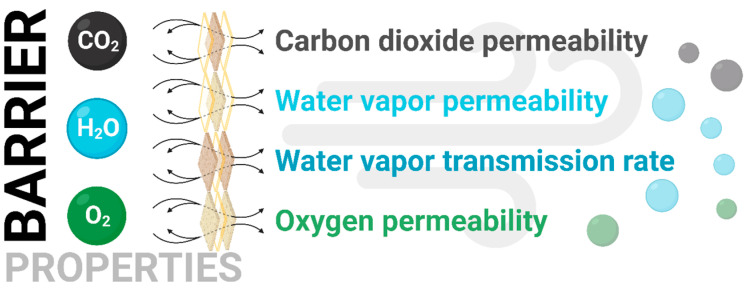
Illustration showing examined carbon dioxide, water vapor, and oxygen barrier properties.

The permeability (Figure 3) of oxygen and carbon dioxide was measured using isobaric equipment. Samples with a surface area of 60 mm^2^ were prepared and placed into the apparatus, where they were exposed to gas at a specified flow rate and pressure. The permeability was described using the following formula:

(4) $$P=\frac{\dot{V}\cdot l}{S\cdot \Delta p}$$

where

V—volumetric flow [mL·s^−1^].
l—the thickness of the sample [m].
S—the area of the sample [m^2^].
Δp—the pressure difference between both sides of the sample [Pa].

Next, the water vapor permeability (WVP) and water vapor transmission rate (WVTP) were calculated using the Aguirre-Loredo method [16]. Samples with a diameter of 95 mm were placed on metal cells that had been previously filled with silica gel. The cells were placed in a desiccator, at the bottom of which was a solution of barium chloride. The water vapor permeability was determined using the masses of the individual samples by utilizing a pressure difference of 2854.23 Pa between the silica gel and the opposite side of the tested film. The cells were weighed every 60 min for 5 h and then after 24 h. The formulas used are presented below:

(5) $$WVP=\frac{\left(WVTP\cdot l\right)}{\Delta P}$$

(6) $$WVTP=\frac{\Delta m}{\left(t\cdot S\right)}$$

where

l—thickness of sample [μm].
Δp—pressure difference [Pa].
Δm—difference in mass of samples [g].
t—time [s].
S—area of sample [mm^2^].

### 2.8. Morphology

Scanning electron microscopy (SEM) analysis was performed using a Zeiss Supra 35 microscope (Carl Zeiss AG, Oberkochen, Germany) equipped with an energy-dispersive X-ray (EDS) detector, enabling detailed structural examination of the prepared films. Images were captured at various magnifications, ranging from the lowest possible setting up to 10.00 KX, on both sides of the examined samples and cross-sections.

### 2.9. UV-VIS Spectra

The obtained film’s light absorption capability in the ultraviolet and visible light range was also tested using the UV-VIS analytical technique. For the measurements, a modern Jenway Genova Bio LifeScience spectrophotometer measuring 198–800 nm with a xenon lamp as the light source was used.

### 2.10. Thermal Analysis

DSC measurements were taken with the use of a Netzsch Polyma 214 calorimeter. Pure substances and nanofibers were measured in Al crucibles under an argon atmosphere. Initially, samples were cooled down to −700 °C, and then samples were measured up to 1500 °C with a 10 K/min heating rate.

### 2.11. Biodegradability Test

To assess hydrolytic degradation, film samples composed of chitosan and its blends with either PCLm or PCLt were incubated in phosphate-buffered saline (PBS) at 37 °C to simulate physiological conditions. Before immersion, each sample was carefully weighed. After 12 h of incubation, the samples were removed, dried at 100 °C, and reweighed. This procedure was repeated at 24, 36, 48, and 72 h to monitor mass loss over time [17,18,19].

## 3. Results and Discussions

### 3.1. Mechanical Properties

**Figure 4:**
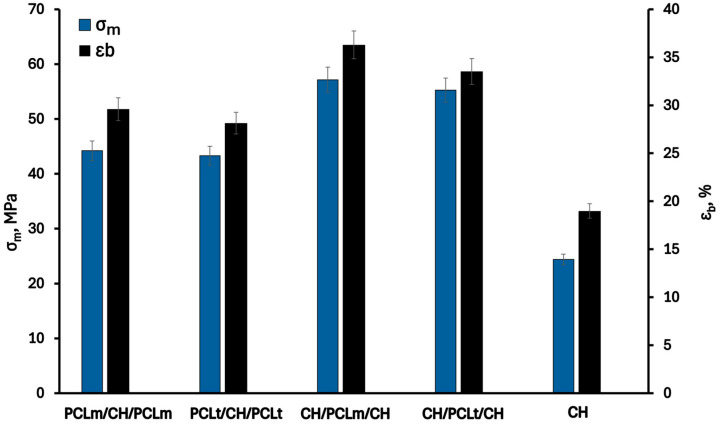
Tensile strength (σ_m_) and elongation at break (ε_b_) of neat chitosan and trilayer chitosan/poly (caprolactone) films with different layer combinations.

Figure 4 shows the mechanical properties of the investigated trilayer films: elongation at break (ε_b_) and tensile strength (σ_m_). As can be seen, the values of tensile strength and elongation at break of the pristine chitosan film are 24.4 MPa and 19.0%, respectively. Each modification resulting in the formation of three-layer films contributes to increased material strength—both in terms of elongation at break and tensile strength—as also confirmed in the literature [20]. The improved mechanical properties can be attributed to factors at both the structural and molecular levels. The electrospun PCL mat, positioned between the chitosan layers, enhances flexibility and energy absorption due to its porous architecture [21]. At the same time, the chitosan layers provide stiffness and surface functionality [22]. Therefore, combining PCL with chitosan layers outside in multilayer films enhances the mechanical and hydrophilic properties of the resulting materials, making them more suitable for the food packaging industry. According to Cooper et al. [23], this phenomenon may be related to physical interactions, such as intermolecular hydrogen bonding between the carbonyl group of PCL and the hydroxyl or amino groups of chitosan, resulting in increased plasticity and reduced stiffness of the material. Furthermore, the partial migration of glycerol and water molecules from the chitosan layer to the PCL mat can also influence the improved mechanical properties of the investigated trilayer films [24].

Considering the arrangement of individual layers, better mechanical performance is observed for films in which the PCL layer is positioned between chitosan layers. The highest values of σ_m_ and ε_b_, equal to 57.1 MPa and 36.3%, respectively, were obtained for the CH/PCLm/CH film, which is more than twice the values recorded for the neat chitosan film. This improvement is attributed to stronger interfacial adhesion in this configuration compared to structures where PCL layers are placed on the outside. The relationship between film structure and mechanical properties was also reported by Tampau et al. [25], who investigated multilayer films composed of internal PCL mats and external starch layers. They observed that the reduction in film cohesion force in the interfacial region decreased stiffness and elongation at break.

### 3.2. Hydrophilic Properties

**Figure 5:**
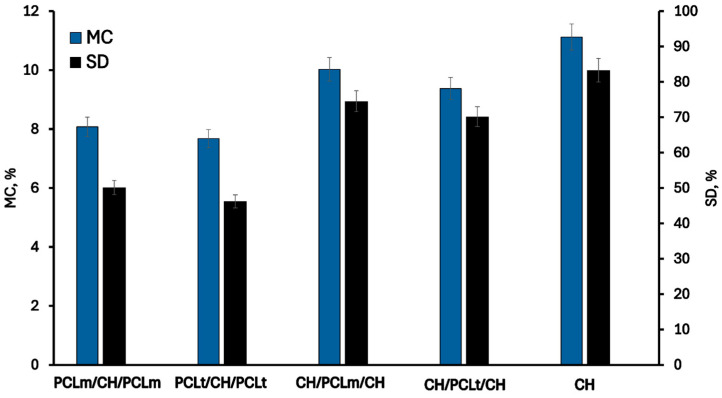
The moisture content (MC) and swelling degree (SD) of the neat chitosan and trilayer chitosan/poly (caprolactone) films with different layer combinations.

**Figure 6:**
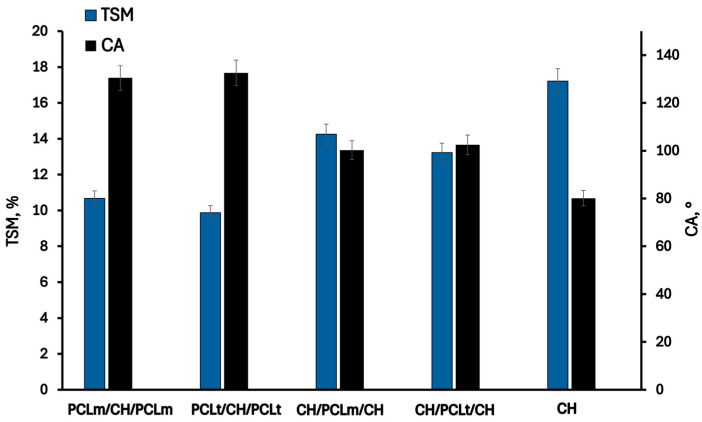
The total soluble matter (TSM) and contact angle (CA) of the neat chitosan and trilayer chitosan/poly (caprolactone) films with different layer combinations.

The changes in hydrophilic properties of chitosan films with PCL fibers are presented in Figure 5 and Figure 6. As can be noticed, the pristine chitosan film is the most hydrophilic. The hydroxyl and amino groups of chitosan interact with the surrounding water, forming hydrogen bonds that play a significant role in achieving good surface wettability [26]. The moisture content, degree of swelling, total soluble matter, and contact angle are 11.13%, 83.31%, 17.21%, and 80.11%, respectively. The addition of poly(caprolactone) (PCL) as a layer to the chitosan mat reduces the hydrophilicity of the films due to the inherently hydrophobic nature of PCL [27]. This hydrophobicity arises from PCL’s chemical structure, which has limited interaction with water. As expected, films with PCL layers positioned on the outer surfaces exhibit lower hydrophilicity and improved moisture resistance. Slightly reduced values of moisture content (MC), water sorption degree (SD), and total soluble matter (TSM), equal to 7.68%, 46.25%, and 9.87%, respectively, were observed for films containing PCLt. This may be attributed to the more compact and irregular structure of electrospun PCLt fibers, which exhibit visible surface defects and denser fiber packing, as revealed by SEM images. These morphological characteristics likely hinder water penetration and decrease overall water uptake. Furthermore, the surface of PCLt/CH/PCLt and PCLm/CH/PCLm films is hydrophobic, with contact angles of 132.53° and 130.45°, respectively. Similar results were reported by Yee Foong & Sultana [28]. The presence of the PCL mat also influences the surface properties of CH/PCLt/CH and CH/PCLm/CH films. The measured contact angles are 102.45° and 100.23°, respectively, indicating that the film’s surface also acquires hydrophobic properties. From the perspective of using these materials in food packaging applications, such hydrophobicity is a desirable feature that enhances their functional performance.

### 3.3. Gas Permeability

**Figure 7:**
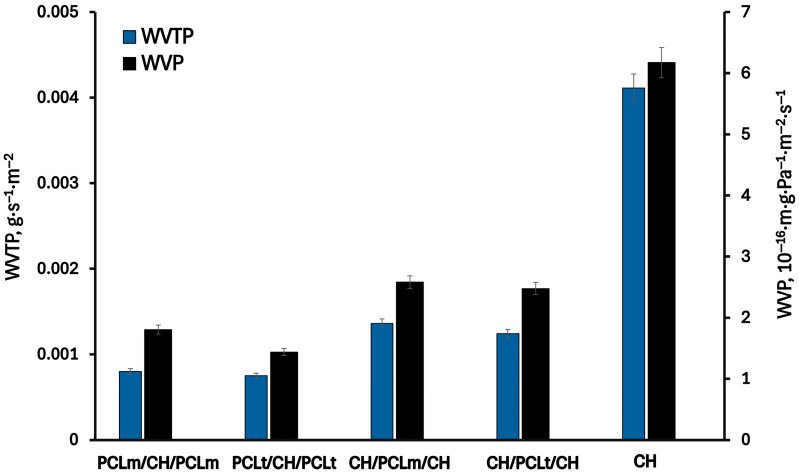
The water vapor transmission rate (WVTR) and water vapor permeability (WVP) of the neat chitosan and trilayer chitosan/poly (caprolactone) films with different layer combinations.

**Figure 8:**
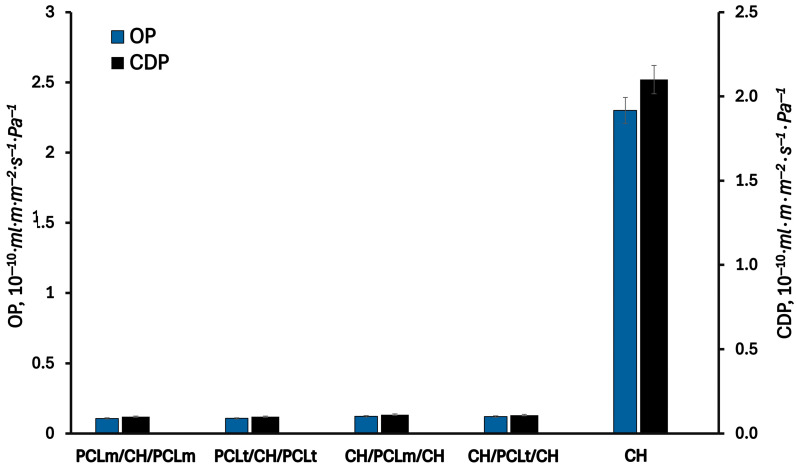
The oxygen (OP) and carbon dioxide permeability (CDP) of the neat chitosan and trilayer chitosan/poly (caprolactone) films with different layer combinations.

Figure 7 and Figure 8 present the results of the permeability of water vapor, oxygen, and carbon dioxide through the investigated films. As can be seen, the permeabilities of WVP, OP, and CDP of the pure chitosan film are equal to 6.2·10^−16^ m·g·Pa^−1^·m^−2^·s^−1^, 2.3·10^−10^ mL*·*m·m^−2^·s^−1^·Pa^−1^, and 2.1·10^−10^ mL·m·m^−2^·s^−1^·Pa^−1^, respectively. As can be expected, the permeation of water vapor is higher than oxygen and carbon dioxide gases due to the hydrophilic nature of chitosan. The chitosan matrix has a high content of hydroxyl and amino groups, which can interact with water molecules to promote the penetration of water molecules through the film matrix [29]. The measured oxygen and carbon dioxide permeability values are two orders of magnitude lower than those for water vapor. According to the literature [30], the chitosan films exhibit excellent barrier properties against oxygen and carbon dioxide, comparable to those of polyvinylidene chloride, low-density polyethene, and ethylene vinyl alcohol. Considering three-layer films, a significant decrease in the permeability of all investigated gases is observed. This can be attributed to the increased thickness of the three-layer films compared to the single-layer one, which results in longer diffusion paths and, consequently, lower gas permeability. Furthermore, the hydrophobic nature of PCL contributes to reducing the permeability of water vapor through the formed films. As water vapor permeability decreases, a corresponding reduction in oxygen and carbon dioxide permeability is also noted. According to Gavara and Hernandez [31], changes in the oxygen permeability are a function of polymer moisture content. This effect is linked to the formation of water clusters and the molecular competition between water and oxygen for the active sites within the polymer matrix. The lowest values of water vapor, oxygen, and carbon dioxide permeability were reached for PCLt/CH/PCLt film and were equal to 1.4·10^−16^ m·g·Pa^−1^·m^−2^·s^−1^, 2.3·10^−10^ mL·m·m^−2^·s^−1^·Pa^−1^, and 2.1·10^−10^ mL·m·m^−2^·s^−1^·Pa^−1^, respectively.

### 3.4. Films Morphology

**Figure 9:**
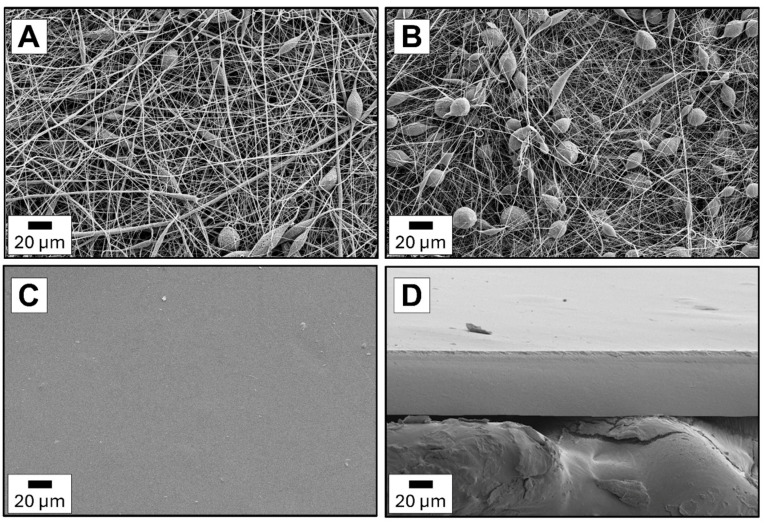
The morphological analysis of electrospun fiber surfaces: (**A**) PCLm, (**B**) PCLt and (**C**) neat chitosan surface and (**D**) cross-section.

**Figure 10:**
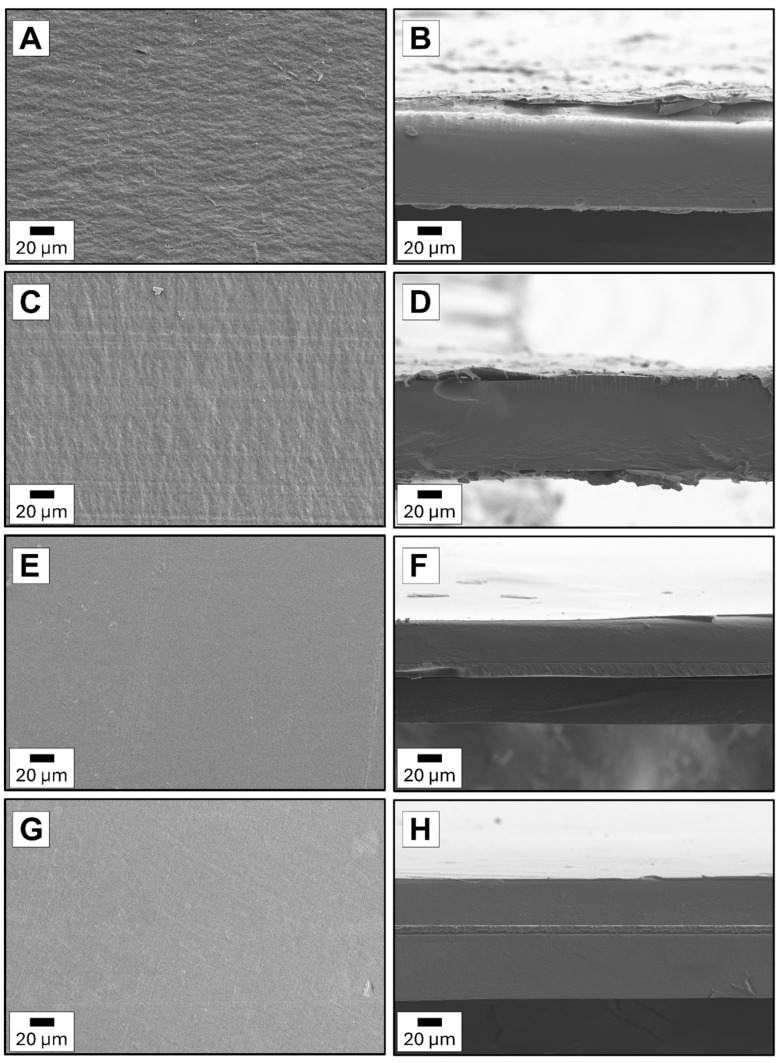
The morphological analysis of trilayer films with different layers combinations of surfaces and cross-sections: (**A**,**B**) PCLm/CH/PCLm, (**C**,**D**) PCLt/CH/PCLt, (**E**,**F**) CH/PCLm/CH, (**G**,**H**) CH/PCLt/CH.

Figure 9A,B shows the images of two types of PCL electrospun fibers: medical and technical. As can be distinguished, the PCLm fibers are more uniform. Defects like branched or splitting fibers and blobs are more visible for PCLt fibers. The diameters of PCLm and PCLt fibers are in the 0.1–2.0 μm range. The pristine chitosan film is smooth without defects on the surface and cross-section (Figure 9C,D). The thickness of this film is 46.5 μm. Considering the multilayer films (Figure 10A–H), it can be seen that the presence of electrospun PCL fibers outside (PCLt/CH/PCLt and PCLm/CH/PCLm) or inside (CH/PCLt/CH and CH/PCLm/CH) chitosan layers. As expected, the thickness of the trilayer films is higher than that of the pristine chitosan film, ranging from 70 to 100 μm.

Furthermore, there is a difference in the surface area and cross-section of films consisting of two PCL layers with a central layer of chitosan compared to films with two layers of chitosan with a central PCL layer. The surface of PCLt/CH/PCLt and PCLm/CH/PCLm is significantly rougher compared to the surface of the CH/PCLt/CH and CH/PCLm/CH films. This is related to the shape of the PCL layer, which is formed from electrospun fibers, making the surface uneven. Considering the cross-section of the investigated trilayer films, it can be observed that the adhesion of all layers to each other is significantly better for films possessing an electrospun fiber layer on the inside. Films with PCL fibers on the outside lacked adhesion, and a gap between the chitosan film and the electrospun PCL mat was observed. The layers were delaminating and detaching from one another. Similar observations were described in Quiles-Carillo et al. [10], where bilayer films obtained after one, two, and three hours of electrospinning deposition of the GA-containing PLA did not strongly adhere to a cast-extruded PLA mat.

### 3.5. UV-VIS Spectra

**Figure 11:**
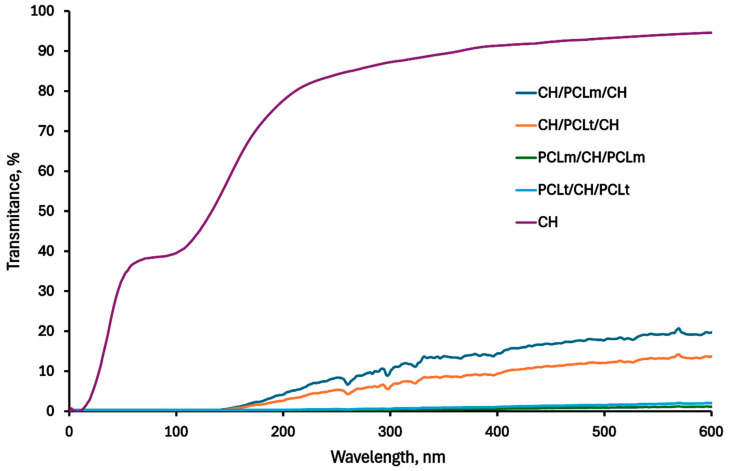
UV-VIS spectra of the neat chitosan and trilayer chitosan/poly (caprolactone) films with different layer combinations.

Figure 11 presents the UV-VIS spectra of pure chitosan and chitosan/polycaprolactone films, illustrating the transparency of the obtained materials. The data show that while both electrospun nanofibers and the addition of multiple layers reduce the overall transparency compared to pure chitosan film, the CH/PCL/CH trilayer films demonstrated notably better transparency than configurations with electrospun nanofibers on the outer layers. In this configuration, PCL acts as an effective bonding layer between the chitosan surfaces, resulting in a more compact and uniform structure that may positively affect light transmittance. This effect is further confirmed by SEM images, which show enhanced cohesion between layers in the CH/PCL/CH films. Additionally, in the Tang and Liu paper [32], a similar phenomenon of electrospun cellulose nanofibers on poly(vinyl alcohol) composite films was confirmed: the higher the percentage of nanofibers used, the lower the transparency of the resulting materials.

In our study, the CH/PCL/CH structure, despite being a trilayer, maintained higher transparency than other configurations, making it particularly advantageous for food packaging applications. The obtained CH/PCL/CH films thus offer an effective combination of structural integrity and transparency, positioning them as a promising material for practical and consumer-friendly food packaging solutions.

### 3.6. Thermal Analysis

**Figure 12:**
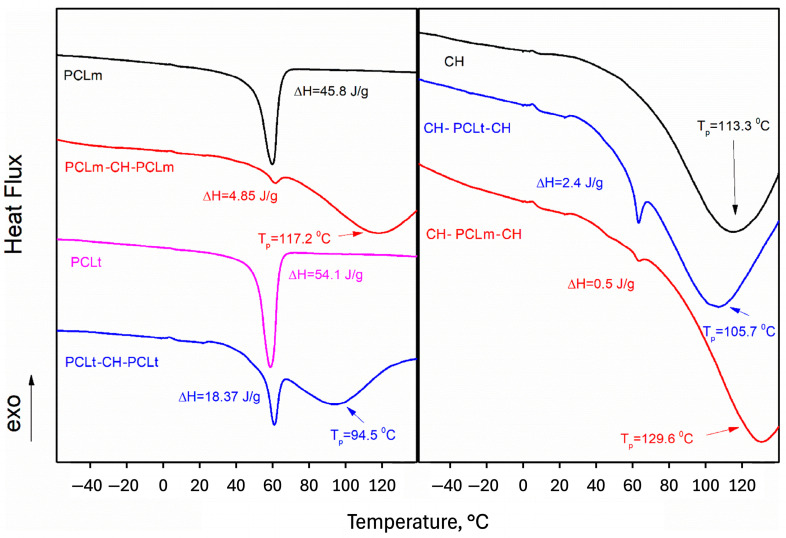
The left panel represents thermograms of measured pure PCL substances (technical and medical) and layered nanofibers PLCt/CH/PCLt and PCLm/CH/PCLm. The right panel represents pure chitosan and layers nanofibers CH/PCLt/CH and CH/PCLm/CH. On both panels, the enthalpy of PCL melting and peak temperatures of chitosan melting were marked.

Calorimetric measurements of pure PCLt and PCLm material showed that the polymers are mainly crystalline (Figure 12). However, the melting enthalpy of medical PCL (45.8 J/g) is 20% lower than that of technical PCL (54.1 J/g), which may indicate that medical PCL contains a higher percentage of amorphous PCL. Calorimetric measurements showed significant differences in molecular interactions between PCL and chitosan layers depending on the type of PCL used (medical or technical). In the case of PCLt, the melting temperature of chitosan is shifted towards lower temperatures, and the melting enthalpy of PCLt is approximately 80% higher than that of PCLm. This behavior is the same in both systems, i.e., PCL/CH/PCL and CH/PCL/CH. The difference in the melting enthalpy of PCL can be attributed to the percentage of amorphous PCL polymer. In contrast, the changes in the melting temperature of chitosan indicate a different strength of interactions. In both the layered systems, the melting temperature of chitosan when medical PCL is used is shifted towards higher temperatures, i.e., 117.2 °C in the case of PCLm/CH/PCLm and 129.6 °C in the case of CH/PCLm/CH systems. This likely indicates stronger interactions between PCL and CH molecules when medical-grade PCL is used. The stronger interactions can be attributed to the higher number of hydrogen bonds or the higher energies of hydrogen bonds in this system.

### 3.7. Biodegradability Test

The hydrolytic degradation of six types of films, pure chitosan (CH), neat PCLm, and PCLt fibers, as well as four multilayer structures (CH/PCLm/CH, CH/PCLt/CH, PCLm/CH/PCLm, and PCLt/CH/PCLt), was evaluated in phosphate-buffered saline (PBS, pH 7.4) at 37 °C to simulate physiological conditions. All tested materials exhibited gradual mass loss over time, with distinct differences depending on their composition and structure. Pure chitosan films degraded the fastest, reaching a mass loss of 20% after 72 h, which is in agreement with previous studies demonstrating the high hydrolytic susceptibility of chitosan in aqueous environments [18,33]. In contrast, neat PCL fibers degraded very slowly, with mass losses of 1.0% for PCLm and 0.6% for PCLt after 72 h [34]. The slower degradation of PCLt may be attributed to the denser and more irregular morphology of the electrospun fibers, which limits water penetration and delays hydrolytic cleavage, as also confirmed by SEM observations.

The multilayer CH/PCL films exhibited intermediate degradation, with mass loss values ranging from 5% to 15% after 72 h, depending on both the type of PCL and the layer arrangement. Films with PCL located on the outer surfaces (PCL/CH/PCL) exhibited lower biodegradability due to the hydrophobic nature of the outer PCL layers, which effectively limited water uptake. In contrast, films with chitosan on the outer surfaces (CH/PCL/CH) showed higher mass loss, as the hydrophilic chitosan facilitates water absorption and enhances hydrolytic degradation. The recorded mass loss values after 72 h were as follows: CH/PCLm/CH: 15.0%; CH/PCLt/CH: 13.2%; PCLm/CH/PCLm: 6.5%; and PCLt/CH/PCLt: 5.0%. These results confirm that the composition and structure of the multilayer films significantly affect their degradation behavior. The placement of chitosan on the surface accelerates degradation, while the presence of hydrophobic PCL on the outside slows the process. Additionally, films containing PCLt showed slightly lower biodegradability compared to their PCLm counterparts, likely due to the more compact fiber structure.

These findings are consistent with previous studies, which have shown that chitosan/PCL combinations enable tunable, composition-dependent biodegradability [35,36]. The observed mass loss confirms the biodegradable nature of the developed multilayer films. It supports their potential use in short-term packaging or coating applications, where moderate durability is followed by environmentally safe degradation.

## 4. Conclusions

In conclusion, the integration of electrospun PCL fibers into chitosan-based films significantly improved their mechanical strength, barrier performance, and functional versatility. Among the tested configurations, CH/PCLm/CH films, with chitosan as the outer layers, demonstrated the most favorable properties, including over twice the tensile strength and elongation at break compared to neat chitosan films. This structure also showed enhanced hydrophobicity, gas barrier properties, and transparency, making it suitable for food packaging applications. SEM and thermal analysis confirmed strong interfacial adhesion, particularly when medical-grade PCL was used. Although technical-grade PCL yielded slightly lower performance, it remains a viable and cost-effective option.

Biodegradability tests confirmed controlled hydrolytic degradation, with degradation rates dependent on layer composition and arrangement. Films with chitosan on the surface showed faster degradation, while PCL surface layers slowed water uptake, offering tunable stability.

Overall, these multilayer biopolymer films offer a promising approach for developing sustainable, high-performance materials with customizable mechanical, thermal, optical, and degradation properties tailored to specific applications.

## Data Availability

The original contributions presented in the study are included in the article, further inquiries can be directed to the corresponding author.
